# Ontology-Driven Search and Triage: Design of a Web-Based Visual Interface for MEDLINE

**DOI:** 10.2196/medinform.6918

**Published:** 2017-02-02

**Authors:** Jonathan Demelo, Paul Parsons, Kamran Sedig

**Affiliations:** ^1^ Insight Lab Department of Computer Science Western University London, ON Canada; ^2^ Purdue Polytechnic Institute Department of Computer Graphics Technology Purdue University West Lafayette, IN United States

**Keywords:** MEDLINE, user-computer interface, information storage and retrieval, medical informatics, PubMed

## Abstract

**Background:**

Diverse users need to search health and medical literature to satisfy open-ended goals such as making evidence-based decisions and updating their knowledge. However, doing so is challenging due to at least two major difficulties: (1) articulating information needs using accurate vocabulary and (2) dealing with large document sets returned from searches. Common search interfaces such as PubMed do not provide adequate support for exploratory search tasks.

**Objective:**

Our objective was to improve support for exploratory search tasks by combining two strategies in the design of an interactive visual interface by (1) using a formal ontology to help users build domain-specific knowledge and vocabulary and (2) providing multi-stage triaging support to help mitigate the information overload problem.

**Methods:**

We developed a Web-based tool, Ontology-Driven Visual Search and Triage Interface for MEDLINE (OVERT-MED), to test our design ideas. We implemented a custom searchable index of MEDLINE, which comprises approximately 25 million document citations. We chose a popular biomedical ontology, the Human Phenotype Ontology (HPO), to test our solution to the vocabulary problem. We implemented multistage triaging support in OVERT-MED, with the aid of interactive visualization techniques, to help users deal with large document sets returned from searches.

**Results:**

Formative evaluation suggests that the design features in OVERT-MED are helpful in addressing the two major difficulties described above. Using a formal ontology seems to help users articulate their information needs with more accurate vocabulary. In addition, multistage triaging combined with interactive visualizations shows promise in mitigating the information overload problem.

**Conclusions:**

Our strategies appear to be valuable in addressing the two major problems in exploratory search. Although we tested OVERT-MED with a particular ontology and document collection, we anticipate that our strategies can be transferred successfully to other contexts.

## Introduction

### Overview and Significance

Seeking information within the published medical literature is important in many domains and contexts [1,2]. Diverse users need to search the literature including physicians [3], medical students [4], cytogeneticists [5], and patients and their relatives [6]. Searches can be roughly categorized into 2 types: *lookup* and *exploratory* [7]. Lookup searches are closed-ended, having precise results and little need for examining and comparing result sets. Exploratory searches, however, are open-ended, having imprecise results and often requiring significant time and effort to work with result sets in order to satisfy the original information need. Examples of exploratory searches with open-ended goals include making evidence-based decisions and updating knowledge to stay abreast of current research findings [2,8]. Although significant progress has been made in supporting lookup searches, exploratory searches are still not well supported, and open-ended search goals are often quite difficult to achieve [2,9,10]. Common barriers to finding relevant medical information include the time it takes to perform searches [3,11], the increasing scope of topical coverage [2], and the information overload that arises from dealing with large result sets [2,3,11-13].

One of the most popular collections of published medical literature is MEDLINE, which comprises more than 25 million documents and is growing every year. The most common means of searching MEDLINE is PubMed, a free search engine and Web interface [14]. Although the search capabilities in PubMed have improved in recent years, there can still be a considerable burden on users when seeking information in the context of exploratory search, due to at least two major problems: (1) the difficulty in articulating information needs using accurate vocabulary and (2) the large number of documents that can be returned from searches. Many users do not have the proper vocabulary to construct effective queries [15,16], which is especially true in medical and health contexts [17-20]. When uncontrolled vocabularies are used, there is no guarantee that concepts are expressed with the same terms in different contexts [13,21]. For instance, if an article contains the term *eye hamartoma*, and a user searches for the vaguer term *eye growth*, there may not be a close match. Thus, without proper terminological knowledge, effective searching can be quite difficult. Adding to the difficulty of searching effectively is the large number of documents that can be returned, which leads to information overload problem [9,22,23]. Dogan et al [2] note that at least one-third of PubMed searches return 100 or more documents. In our own testing, searches for common terms (eg, “breast cancer” or “brain tumor”) returned many thousands of documents.

**Figure 1:**
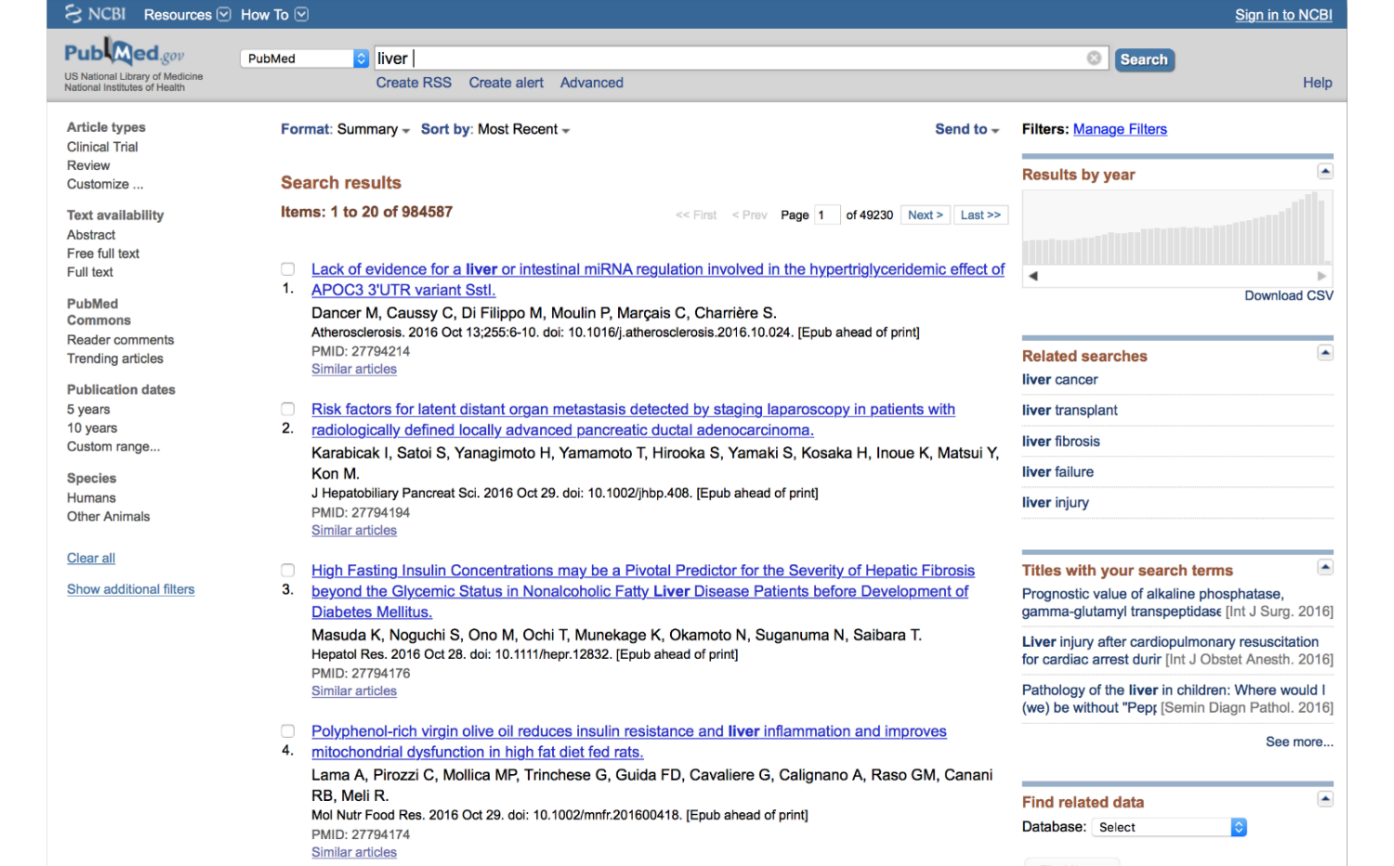
A screenshot of PubMed showing results from searching for “liver.”

Interfaces to most search engines, including PubMed, use simple text boxes into which users enter query terms. This interface style does not assist users in articulating their information needs [24] and works well only for lookup search tasks [25,26]. For example, if a user is interested in finding information about “liver,” but is not sure what terms are relevant in articulating a query, he or she must simply enter “liver” into the search box. As the query is vague, a very large set of documents is returned—almost one million documents spanning over 4900 pages when using PubMed (Figure 1).

Multiple strategies have been employed to help support query formation in exploratory search contexts by replacing the standard text box, including faceted search [27], visualization widgets [28], query previews [29], and hierarchical presentation of expansion terms [30]. The common theme among these strategies is that meaningful information is extracted from the document collection and then represented in a manner that can help the searcher recognize terms that will more accurately describe the information they are seeking. Such strategies promote recognition over recall, not relying on users having to know and retrieve correct vocabulary from memory [24].

We present Ontology-Driven Visual Search and Triage Interface for MEDLINE (OVERT-MED), a Web-based visualization tool that addresses two major difficulties in searching large document collections: (1) the difficulty in articulating information needs with useful vocabulary and (2) the difficulty in dealing with large search result sets. To address the first difficulty, we propose the idea of using a formal ontology to help users build domain-specific knowledge and vocabulary. To test this, we have implemented a searchable index of the Human Phenotype Ontology (HPO) that provides users with suggestion terms that are related to their information needs. To address the second difficulty, OVERT-MED supports multistage interactive triaging of search results using interactive visualization techniques. We use a custom-built index of MEDLINE, which comprises approximately 25 million documents, as our searchable collection of medical literature. Although OVERT-MED has been initially developed for use with a particular ontology and document collection, we expect that our design ideas will transfer to other contexts. The following subsections provide background information and discuss related work.

### Ontologies

One way to meaningfully extract and model information from a domain is to construct an ontology [31,32]. An ontology represents concepts and their relationships using a standard vocabulary [32]. Ontologies serve many practical functions, including clarifying the structure of knowledge within a domain, providing a common vocabulary, enabling computational analysis, and supporting knowledge sharing [31-33]. Ontologies often capture concepts within a domain at multiple levels of abstraction. For instance, an anatomy ontology may have a concept *body*, a sub-concept *face*, a further sub-concept *nose*, and so on. The concepts in an ontology can be represented using many different structures, including trees and different types of graphs.

The ontology we are using, HPO, has been curated by domain experts in an attempt to capture all phenotypic abnormalities that are commonly encountered in human monogenic disease [34]. In our previous work with genomics researchers, we learned of the importance of HPO in their workflow, including in activities involving literature search [5]. HPO is widely used in the biomedical field, is regularly updated, and has a high level of quality control. It is also available for download in the popular Open Biomedical Ontologies (OBO) and Web Ontology Language (OWL) formats. For these reasons, we believe HPO is ideal for testing our proposal of using ontologies to address the vocabulary problem. It should be noted that we are not suggesting HPO is better than other ontologies or that it should be used in all contexts. HPO is only one of the many ontologies that could be used to support exploratory search, and search systems should make use of whichever ontologies are most appropriate for given contexts.

### Document Triage

Triaging is an activity that involves determining the relevance of documents to an information need [35]. Triaging activities are often time-constrained and require quick assessment of relevance with incomplete knowledge. For example, a search may return hundreds or thousands of potentially relevant documents. As it is not feasible to read each one in detail, users must sort through the documents and quickly assess their relevance based on incomplete knowledge of their contents. Research suggests that triaging takes place in 3 successive stages: (1) the “multiple document” stage, where initial relevance judgments are made to select documents from a set without careful examination; (2) the “individual document” stage, where individual documents are examined in more detail and categorized (eg, kept or rejected); and (3) the “further reading” stage, where a small set of documents are read in depth to extract relevant information and satisfy the original information need [36]. In addition, research shows that triaging often occurs in a cyclical and iterative fashion, where the above stages are revisited multiple times [37].

### Search Result Visualization

Most search interfaces present results in a traditional list-based manner, where documents are ranked and textually represented using a title and various metadata. While not a problem for simple lookup search tasks, traditional list-based representations are not effective in supporting exploratory search tasks, which are typically open-ended and involve complex information needs [38]. Although lists are familiar and simple, studies show that users rarely examine lists fully or carefully [39] and seldom venture past the first few pages of results [40]. Scanning through long lists can be tedious and cognitively demanding. Visualizations of search results can overcome some of the problems associated with textual list-based representations by shifting cognitive burden onto the perceptual system. For instance, whereas visualizations can be scanned freely by the eyes, text must be scanned sequentially, requiring more time and cognitive effort to detect patterns and relationships [41,42]. In addition, visualizations can encode a significant amount of information within a small space, removing the need to navigate multiple pages to view search results. Previous work has demonstrated the utility of visualizations in document search, exploration, and analysis [43,44].

### Related Work

Some researchers have recognized the value of using ontologies to better support search activities (eg, [13,45]). The central focus of this research is term extraction and mapping, which is done using text mining and natural language processing techniques. In this body of work, ontologies are used to improve search performance computationally without involving users. The fundamental difference compared with our work is that we use ontologies to help users develop knowledge and domain-specific vocabulary—that is, the focus is on the user rather than on algorithms and other computational processes. Our approach is important in contexts where users have valuable knowledge and context-specific goals that cannot be replaced by computation—in other words, users need to be kept “in the loop.”

Other researchers have focused on developing interfaces to MEDLINE as alternatives to PubMed. For example, Wei et al have developed PubTator, a PubMed replacement interface that uses multiple text mining algorithms to improve search results [46]. PubTator also offers some support for document triaging. Whereas PubTator appears interesting and useful, it relies on queries being input into the standard text box, and it presents results in a typical list-based fashion. Thus, it is not aimed at addressing either of the two problems we are attempting to address with OVERT-MED—that is, the vocabulary problem and the information overload problem. Other alternative interfaces that offer interesting features but do not address either of the two problems include SLIM [47] and HubMed [48]. An alternative interface that potentially provides support in addressing the first problem is iPubMed [49], which provides fuzzy matches to search results. An alternative interface that may provide support in addressing the second problem is refMED [50], which provides minimal triaging support through relevance ranking. A for-profit private tool, Quertle, appears to use visualizations to mitigate the information overload problem, although very few details are publicly available. Lu [51] provides a detailed survey that includes many other alternative interfaces to MEDLINE, although none are aimed at solving either of the two problems that we are addressing here.

In summary, no extant research explores the combination of (1) ontologies to help build domain-specific knowledge and vocabulary when users need to be kept “in the loop” and (2) triaging support using interactive visualizations to help mitigate the information overload problem. The following sections provide details about our approach to addressing these issues.

## Methods

### Overview

We developed OVERT-MED to test our proposed solutions to the two problems described hereinbefore. To anchor our research in a specific context, we chose MEDLINE as our document collection. MEDLINE offers an interesting testbed because of its popularity and size. We developed a custom index of MEDLINE so that it can be queried from the front end of OVERT-MED. We have also indexed HPO to help users build knowledge and domain-specific vocabulary.

### Indexing of MEDLINE and HPO

We downloaded the entire MEDLINE database, which has been made freely available by the National Library of Medicine (NLM) for research purposes. The MEDLINE database consists of article “citations,” which are essentially article metadata, including authors, journal title, Medical Subject Heading (MeSH) keywords, publication date, and other fields. Also included in each citation is the abstract text. We developed a custom index using the open-source Apache Solr and Lucene projects. Lucene supports full-text indexing and search functionality, and Solr is a search platform that runs on the Lucene index. To rank documents, Lucene uses the well-known term frequency-inverse document frequency (tf-idf) scheme [52]. Lucene also ranks results based on an internal similarity measure that generates a vector space model (VSM) score [53], using index terms as dimensions and tf-idf values as weights. We have described our indexing strategy in greater detail earlier [5].

HPO is a formal ontology of human phenotypic abnormalities found in human disease [34]. Each entry in HPO describes a phenotypic abnormality such as melanoma or hepatoblastoma. HPO is under active development and currently contains more than 11,000 terms. We have also indexed HPO in our Lucene index. HPO contains multiple fields for each phenotype in the ontology, including name, definition, id, synonyms, and commentary from domain experts. We index all fields to provide robust vocabulary suggestions—when a user enters a term, all fields in the index are examined, which provides much more useful information than would result from looking for only exact matches on the phenotype name. This is described using an example in greater detail in the following.

### Development and Architecture

**Figure 2:**
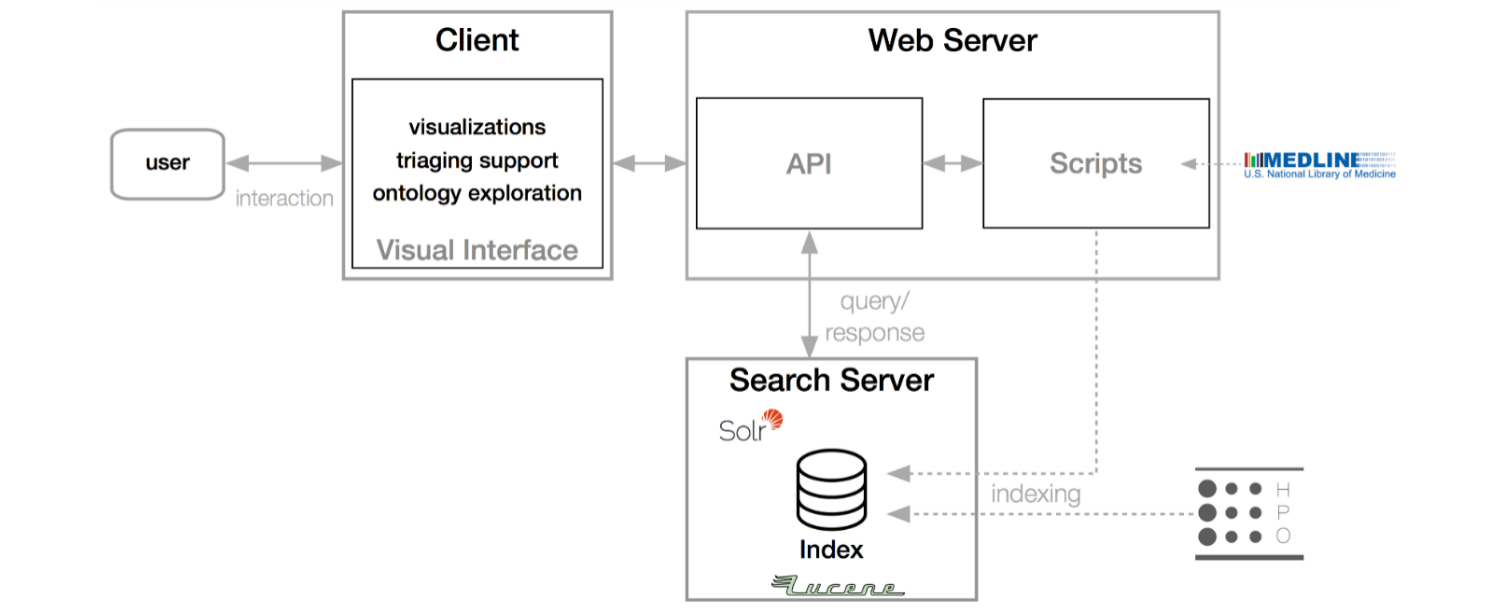
Client-server architecture of the Ontology-Driven Visual Search and Triage Interface for MEDLINE (OVERT-MED) system.

We developed OVERT-MED as a Web-based tool that runs in any modern browser. It connects to a Web server that stores our indices and handles search requests (via our Solr search server). We have developed a series of scripts to retrieve MEDLINE updates from the NLM public ftp site and to construct the indices for MEDLINE and HPO in our Lucene index. We have also developed an application programming interface (API) that handles requests for searches and other basic functions. The front-end has been developed using HTML5, CSS, and JavaScript. The visualizations have been developed using D3.js [54], a popular JavaScript visualization library. Figure 2 provides a diagrammatic overview of the architecture of the OVERT-MED system.

## Results

### Ontology Term Suggestion

**Figure 3:**
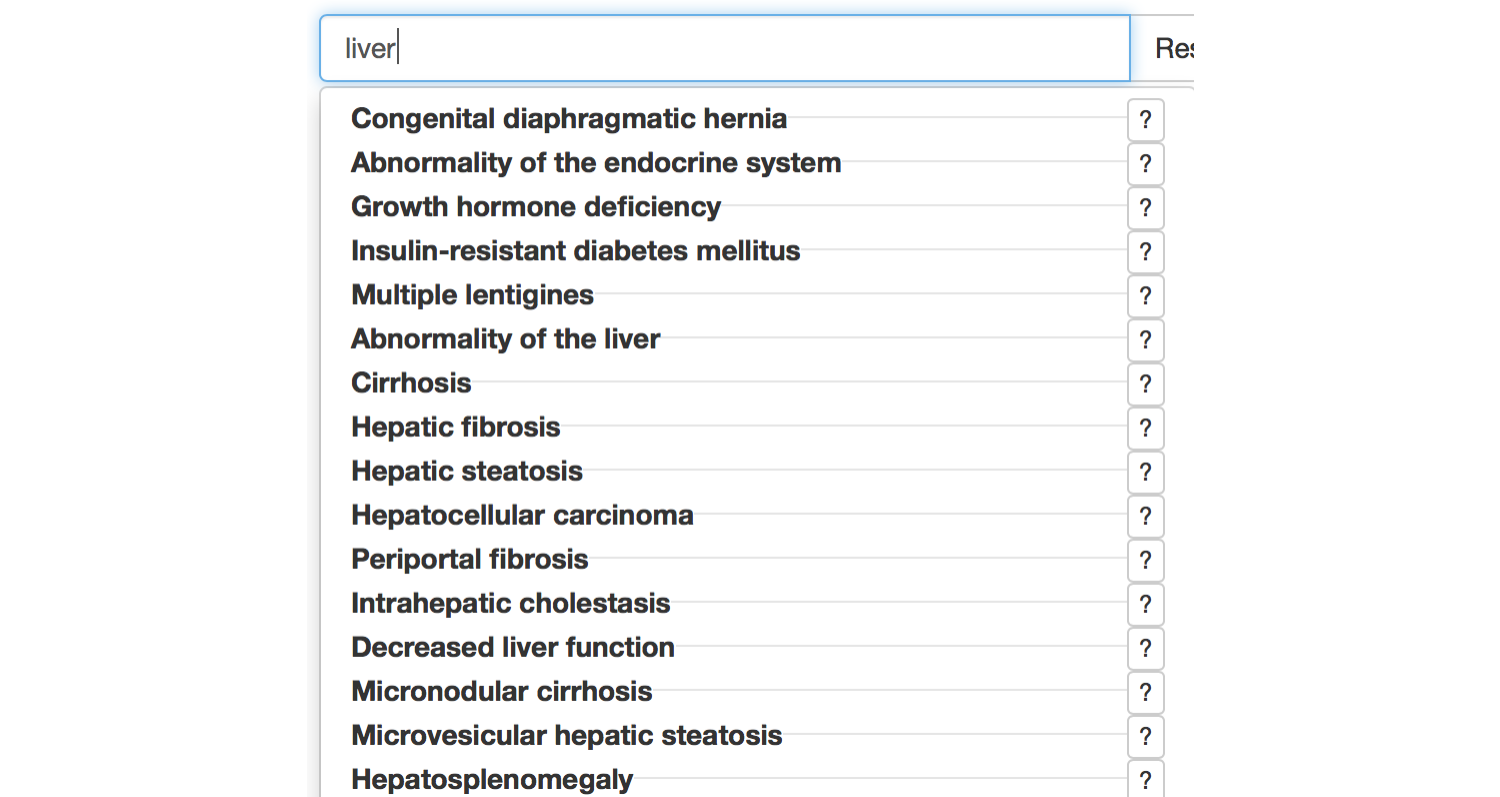
The ontology term suggester, showing results from typing “liver.”

OVERT-MED uses HPO to help users better articulate their search needs through a technique we call *ontology term suggester*. Users enter terms into a text box, and a set of suggestions (phenotypes) are provided. The suggestions are updated in real-time as a user types each character. In addition, to providing better terminological support, we look for matches on both the phenotype names as well as descriptions and expert commentary on the phenotypes (these are not shown to users, but are indexed on our server). For example, a user may be interested in finding articles related to the term “liver,” but may not have sufficient vocabulary to articulate a useful query involving relevant terms. Figure 3 shows the ontology term suggester after typing “liver” into the search box. Phenotypes related to the liver are displayed. Results such as “Growth hormone deficiency” and “Ascites” are displayed because they have a connection to the liver—the effects of growth hormone are mediated by insulin-like growth factor, which is produced primarily in the liver; and ascites is commonly associated with liver disease. Many of the returned phenotypes do not have the term *liver* in their name, but are related to the liver. In a traditional search interface, there is no way for a user to get from “liver” to “ascites” or “growth hormone deficiency.” Finally, because users may not understand a particular phenotype (eg, congenital diaphragmatic hernia), selecting the “?” button will open a new tab and load the official entry in the HPO Web browser. From there users can find more details, including associated genes and diseases. This search strategy can help users build knowledge of the domain and vocabulary that can be used to enhance cognitive performance and exploration.

### Sensitivity Encoding for Query Refinement

A well-known problem in open-ended search tasks is that potentially relevant results may not be displayed if they do not meet the specified search criteria. For example, when searching for a house to buy, users often have ill-formed criteria, such as price range, number of bedrooms and bathrooms, yard size, location, and so on. Although certain search criteria may be specified (eg, 4 bedrooms, under $200,00), results that do not meet the criteria may also be relevant, such as a house that has only 3 bedrooms but is a great price. When using visualizations to support such search tasks, certain criteria can be relaxed and results that do not meet certain criteria can be visually encoded in different ways. For instance, results that do not meet number of bedrooms can be encoded with 1 color; results that do not meet yard size can be encoded with another; and so on. Visually encoding this type of information can provide cues to users to adjust their search criteria so that potentially relevant results are included. This visualization strategy, known as sensitivity encoding, has been shown to be beneficial in a number of contexts [55,56].

**Figure 4:**
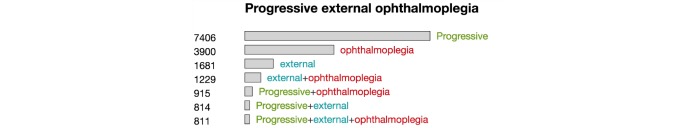
A set of sensitivity-encoded query selectors for “progressive external ophthalmoplegia.”

**Figure 5:**
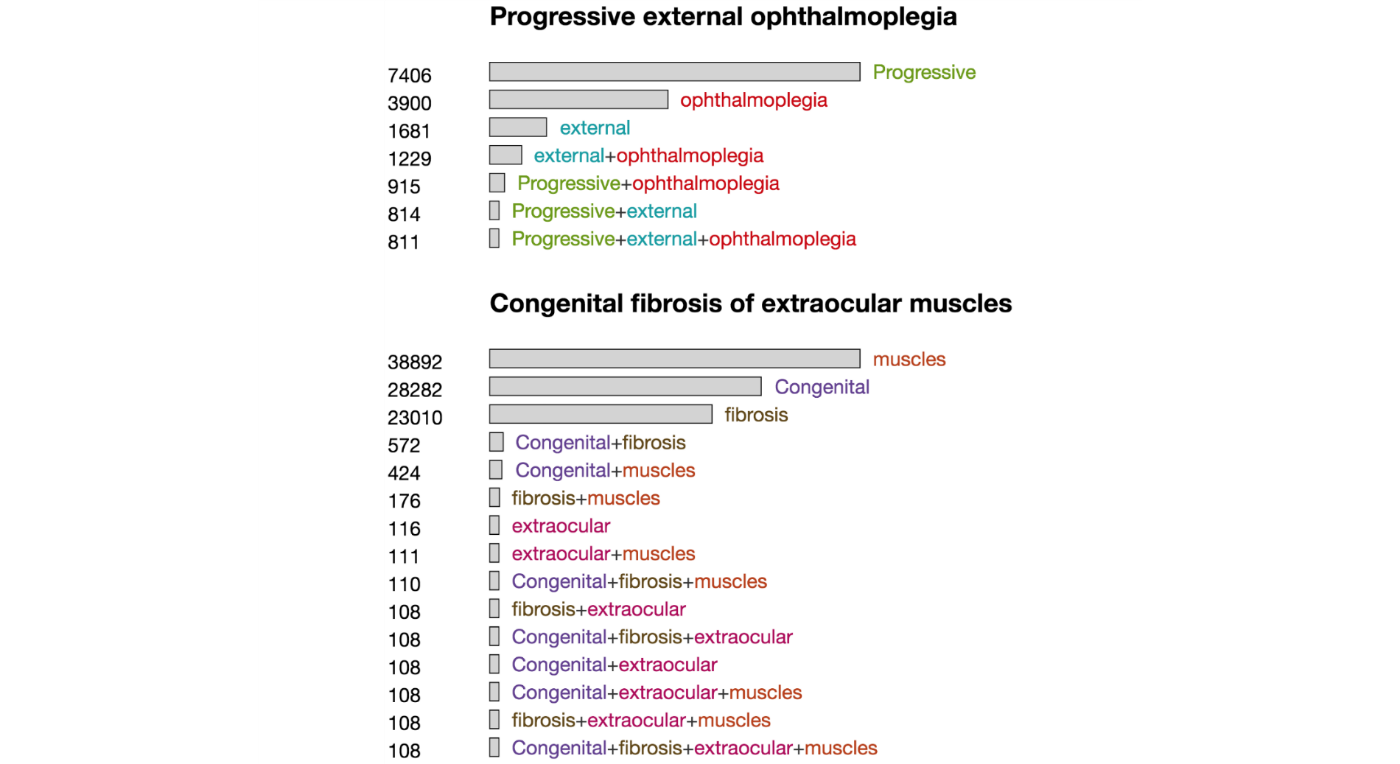
The result of adding a second phenotype via the ontology term suggester, which leads to more sensitivity-encoded query selectors.

Although OVERT-MED supports the selection of precise phenotype names, the exact combination of words in a name may be too restrictive, and may not provide the most relevant results. For example, a user may select the phenotype *progressive external ophthalmoplegia*. Our index shows 811 articles associated with this specific phenotype. However, users may be interested in articles associated with different variations of the words—for example, *progressive opthalmoplegia* or *external opthalmoplegia.* We use a set of *Sensitivity Encoded Query Selectors* in OVERT-MED to handle this issue. When a phenotype is selected, we perform searches on our index using all possible combinations of the words and then visually encode the size of the result set. Figure 4 shows the result of a user selecting “progressive external opthalmoplegia.” The number of matching articles for each combination is provided numerically and encoded visually using the length of the bar next to each combination. From Figure 4, we can see that if the user relaxes the term to “progressive ophthalmoplegia,” an additional 104 articles show up in the index and with “external opthalmoplegia,” an additional 418 articles show up. Without such a sensitivity encoding strategy, many of these potentially relevant results would not be made available. As users are often interested in more than 1 phenotype, multiple phenotypes can be selected, each of which is subjected to the same sensitivity encoding process. Figure 5 shows a second phenotype, congenital fibrosis of extraocular muscles, being added.

### Interactive Triaging Support to Mitigate Information Overload

**Figure 6:**
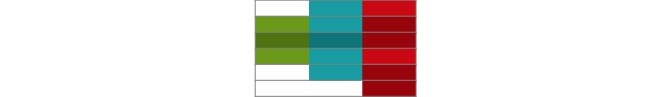
The query result heatmap: 6 documents are represented by 6 rows, where each column represents a term (progressive external opthalmoplegia).

OVERT-MED provides multistage triaging support to mitigate the information overload problem. Multiple design strategies support the first stage of triaging—the “multiple document” stage. First, when a specific set of terms is chosen, the metadata from up to 250 documents are visualized. Each document is encoded using a small bar, and the presence of each term is encoded using a section of the bar. Figure 6 shows how 6 documents are represented in the case of 3 terms (progressive external opthalmoplegia). Within the visualization, each row represents a document, and each column represents one of the phenotype words. The words are color coded—in this case, green for progressive, teal for external, and red for opthalmoplegia. A white cell indicates no occurrence of the word. The visualization functions as a type of heatmap [57], where the color saturation encodes the frequency of a term within a document. We call this technique the *query result heatmap.* In Figure 6, a darker red means higher occurrence of the word opthalmoplegia. This type of encoding can aid in rapid visual scanning and identification of potentially relevant documents [43,58].

**Figure 7:**
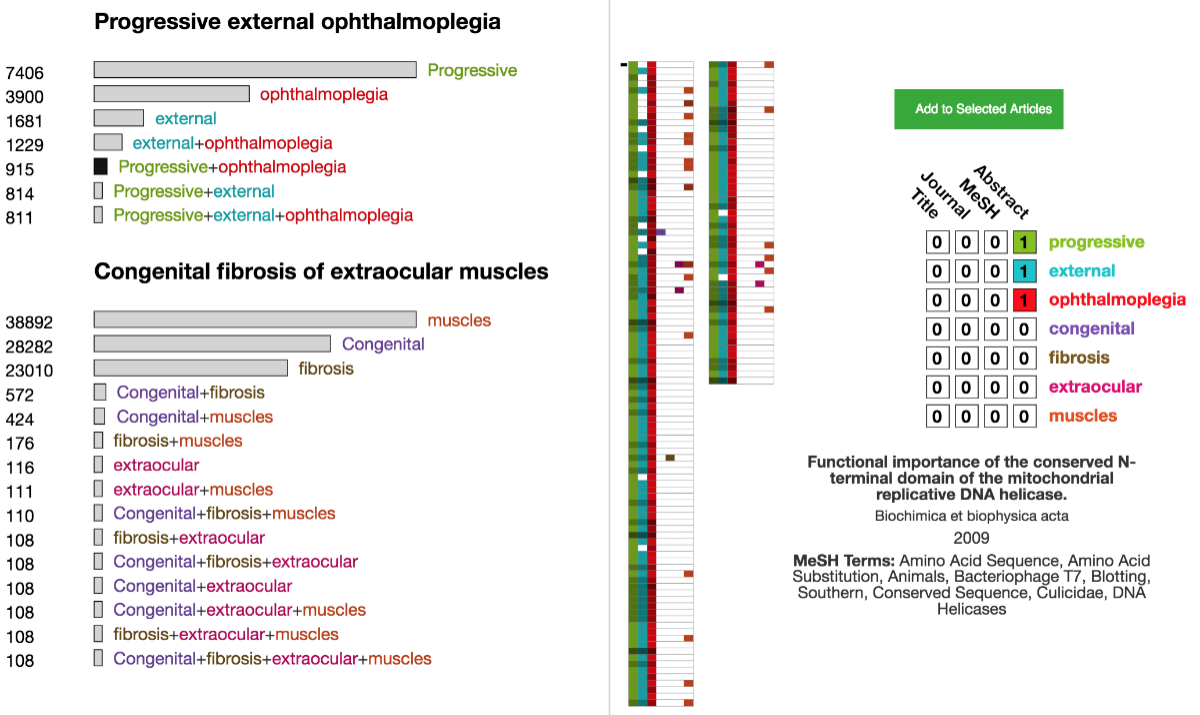
State of the interface after a user has selected “progressive+opthalmoplegia.”

To further support the triaging activity, OVERT-MED allows users to interactively explore metadata associated with the matching documents. Figure 7 shows the state of the interface after a user has selected “progressive+opthalmoplegia.” The first 250 documents (ranked by our indexing algorithm) are encoded in the Query Result Heatmap. Each row functions as an individual document heatmap, showing the occurrence of the 7 phenotype terms within the document. Because the user has selected “progressive” and “opthalmoplegia,” all documents indicate occurrences of both terms. It is readily apparent that most of the documents also contain the term “external.” Approximately 20 also contain “muscles,” 4 contain “extraocular,” 1 contains “fibrosis,” and 1 “congenital.”

**Figure 8:**
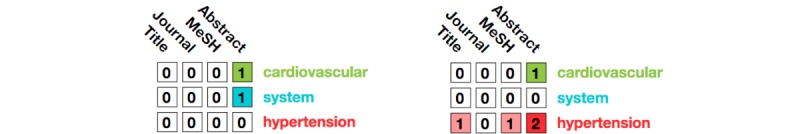
The term distribution matrix for 2 different documents within the same result set.

OVERT-MED also provides a *Term Distribution Matrix* to help users quickly determine document relevance while browsing the Query Result Heatmap. Within the term distribution matrix, users can see the occurrence of terms in 4 places within the document metadata: (1) title, (2) journal name, (3) MeSH terms, and (4) abstract text. The document title, journal, year, and MeSH terms are also displayed. This representation helps users make decisions about relevance via quick visual scanning. For example, if a term appears only in the journal name it may not be very relevant, but if a term appears 5 times in the abstract text it is more likely to be relevant. Users can perceive this type of information quickly due to the categorical color encodings. Figure 8 shows the term distribution matrix for 2 different documents within the same result set. Through rapid visual scanning, even without reading the text, it is apparent that the terms are quite important in the document on the right.

**Figure 9:**
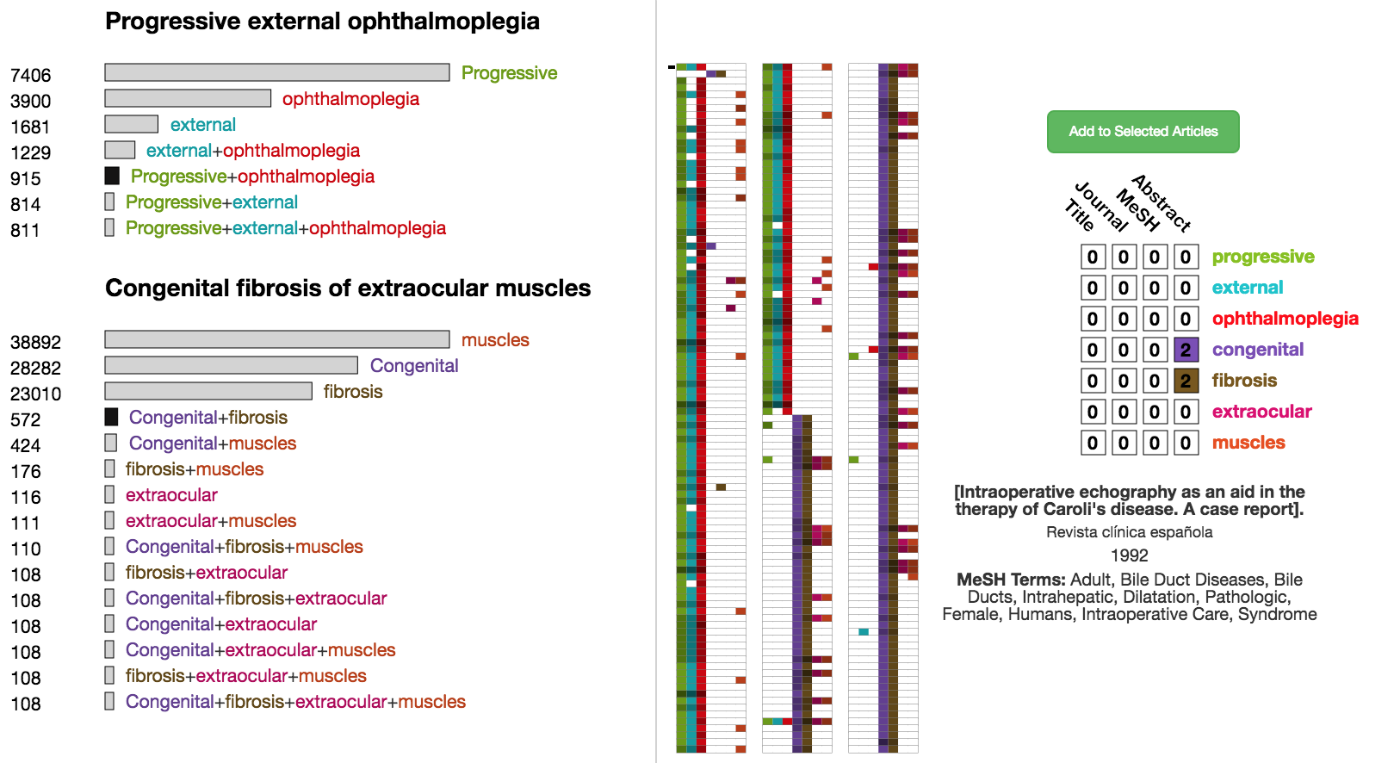
The result of a user adding documents containing “congenital” and “fibrosis” for comparison.

To support rapid exploration—a fundamental goal of triaging—the keyboard arrow keys can be used to move quickly through the documents while the metadata is dynamically updated. If a relevant document is detected, users can hit the “enter” key or click the button to add the document to a pile for subsequent investigation (this stage is explained in greater detail in the following). This stage of triaging also allows for quick comparison of cooccurring phenotypes within documents. For example, Figure 9 shows the result of a user adding documents containing “congenital” and “fibrosis.” It is immediately clear through quick visual scanning that not many documents contain both “congenital fibrosis” and “opthalmoplegia.”

**Figure 10:**
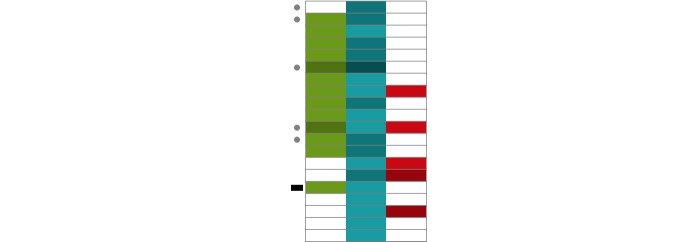
Closeup view of the query result heatmap.

While browsing the query result heatmap, it may be difficult to remember which documents have been visited previously. This is especially true in the context of iterative triaging, where users may return to the heatmap after being away for some time. In OVERT-MED, when users pause on a document for 5 s or more, a small mark is placed beside the document to serve as a visual reminder (Figure 10). When revisiting the heatmap, users can quickly recognize which documents they have previously examined. We assume that 5 s is a reasonable threshold for determining when a user has examined the *term distribution matrix*.

**Figure 11:**
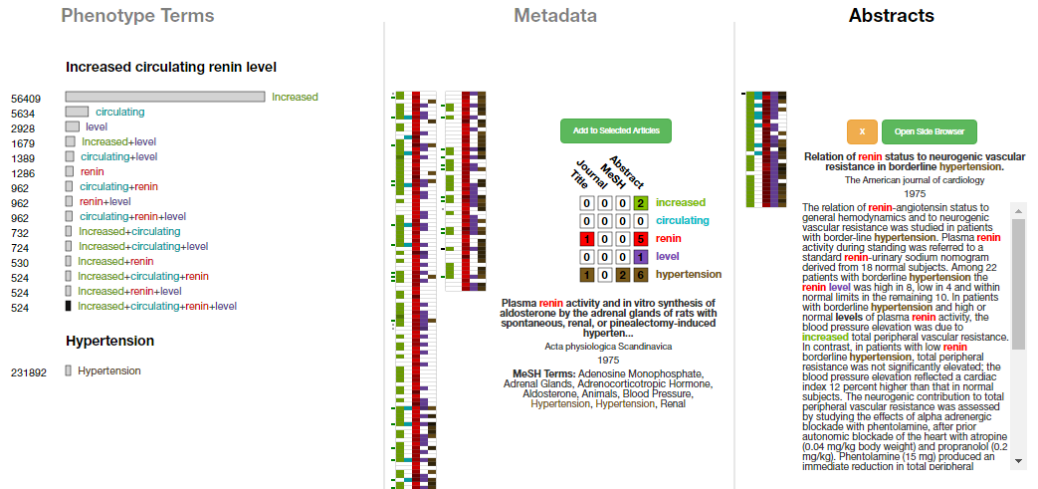
Twenty-nine documents have been selected to examine in closer detail.

The next stage in the triaging activity—the “individual document” stage—involves examining individual abstracts of previously chosen articles. At this stage, users are likely to have narrowed down the number of documents significantly. Documents are encoded via a *Selected Pile Heatmap* in the same manner as in the query result heatmap, and each can be selected to view its abstract. In this *term-encoded abstract*, matching terms are color coded to facilitate quick identification, especially within the abstract text. Figure 11 shows an example in which the user has selected 29 documents, which are encoded in the selected pile heatmap and the term-encoded abstract is displayed for the first document. Even before reading the text in detail, it is easy to see that “renin” and “hypertension” both appear frequently, indicating that they are important. Thus, users can scan the text quickly to get a sense of the appearance of the query terms, without having to necessarily read the text sequentially. An important aspect of this stage of triaging is the ability to quickly categorize documents. In OVERT-MED, users can quickly reject a paper by selecting the orange “x” button, or can quickly add a paper to the next stage by selecting the green button or pressing the “enter” key.

**Figure 12:**
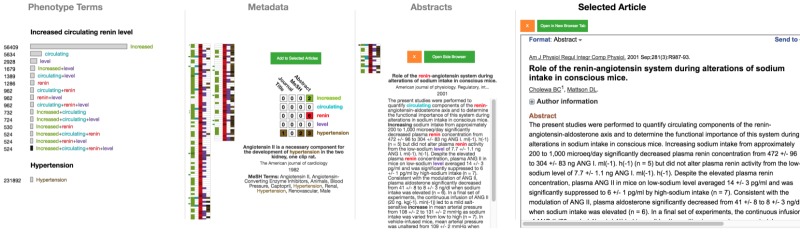
Full-screen capture showing all components of OVERT-MED where a user has traversed all stages of a search and triaging activity. OVERT-MED: Ontology-Driven Visual Search and Triage Interface for MEDLINE.

The final stage of triaging is the “further reading” stage, where a small set of documents are read in-depth to extract relevant information and satisfy the original information need. Although this stage could be supported in various ways, we support this stage in OVERT-MED by presenting a PubMed entry for a selected document in an embedded frame directly within the interface of OVERT-MED. This allows for quick inspection of any PubMed details that are important to the user, such as full-text links, citation details, and PubMed Commons links, and also allows users to login to their *National Center for Biotechnology Information* (NCBI) account to save the article to a collection, compare with other saved articles, and so on. There is also a button to open the PubMed link in a new browser tab if a user needs more space. Figure 12 shows a full-screen capture of OVERT-MED in which a user has traversed all stages of a search and triaging activity.

**Figure 13:**
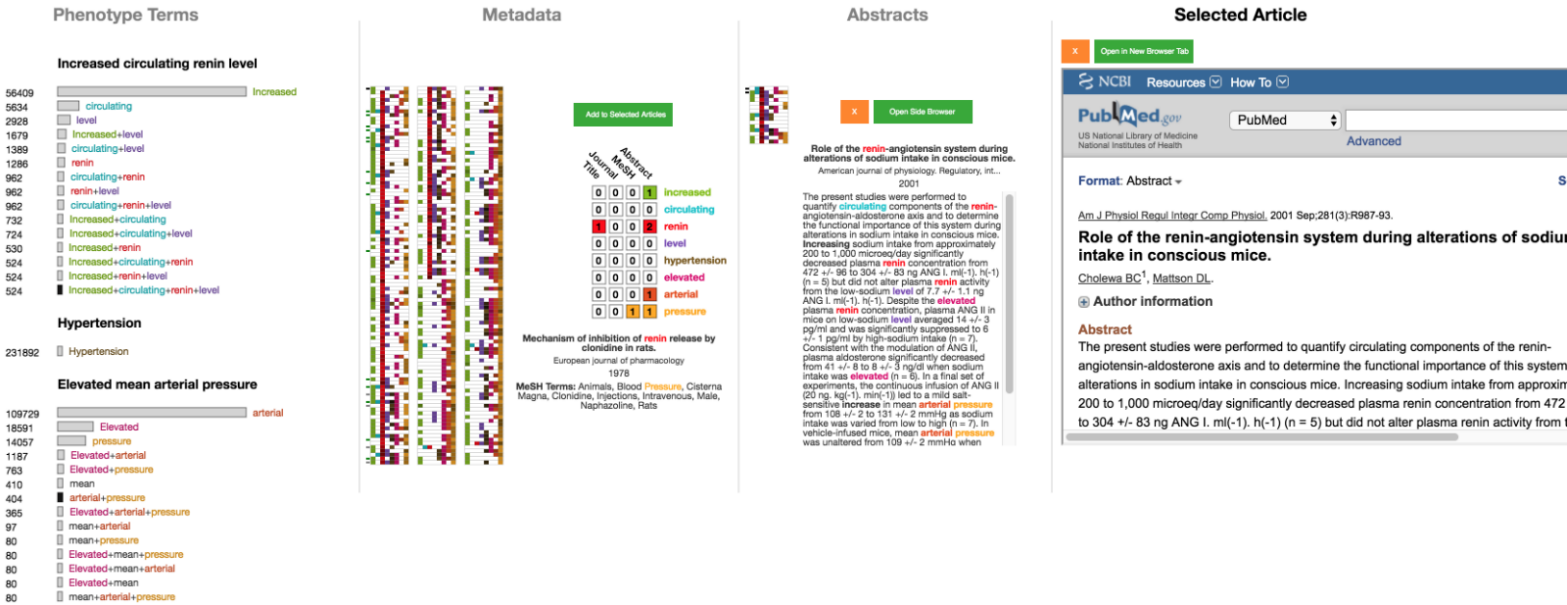
The interface after a user has examined a document in detail in the final stage, discovered a link, and has returned to the initial stage with a new information need.

As research shows that triaging activities are cyclical and iterative, we have designed OVERT-MED to be flexible in this regard. At any point during an activity, users may adjust their query or document selections, and each component of the interface will dynamically reflect any changes. For example, a user may reach the final stage of triaging and find a term within a document that seems relevant to the original information need. The user can return to the initial stage of entering the term and selecting phenotypes. In doing so, the rest of the interface remains stable and the user can proceed through any of the triaging stages. Figure 13 shows the interface after a user has examined a document in detail in the final stage, discovered a link between renin level (the original phenotype of interest) and arterial pressure, and has returned to the initial stage to find a phenotype related to arterial pressure. The user discovers a phenotype named “elevated mean arterial pressure” and selects it. At this stage, the user is not particularly interested in whether the arterial pressure is elevated, and simply wants to explore the relationship between renin level and arterial pressure. Due to our sensitivity encoding strategy, the user can select “arterial+pressure” to add documents with those 2 terms. From this point, the user can continue through the triaging stages or return to the initial stage again.

## Discussion

### Overview

OVERT-MED was developed to address two major problems that are known to exist in complex, exploratory search activities: (1) the difficulty in articulating information needs due to insufficient knowledge and domain-specific vocabulary, and (2) the difficultly in dealing with information overload due to the large number of results returned. To address the first difficulty, we proposed the idea of using a formal ontology to help users build domain-specific terminology and knowledge for constructing search queries. To assist in this process, we indexed HPO and provided a search feature that provides robust results to terms that are entered. To address the problem of search criteria being too restrictive in open-ended contexts, we used a visual sensitivity encoding strategy to help users see possibilities with different combinations of terms.

**Figure 14:**
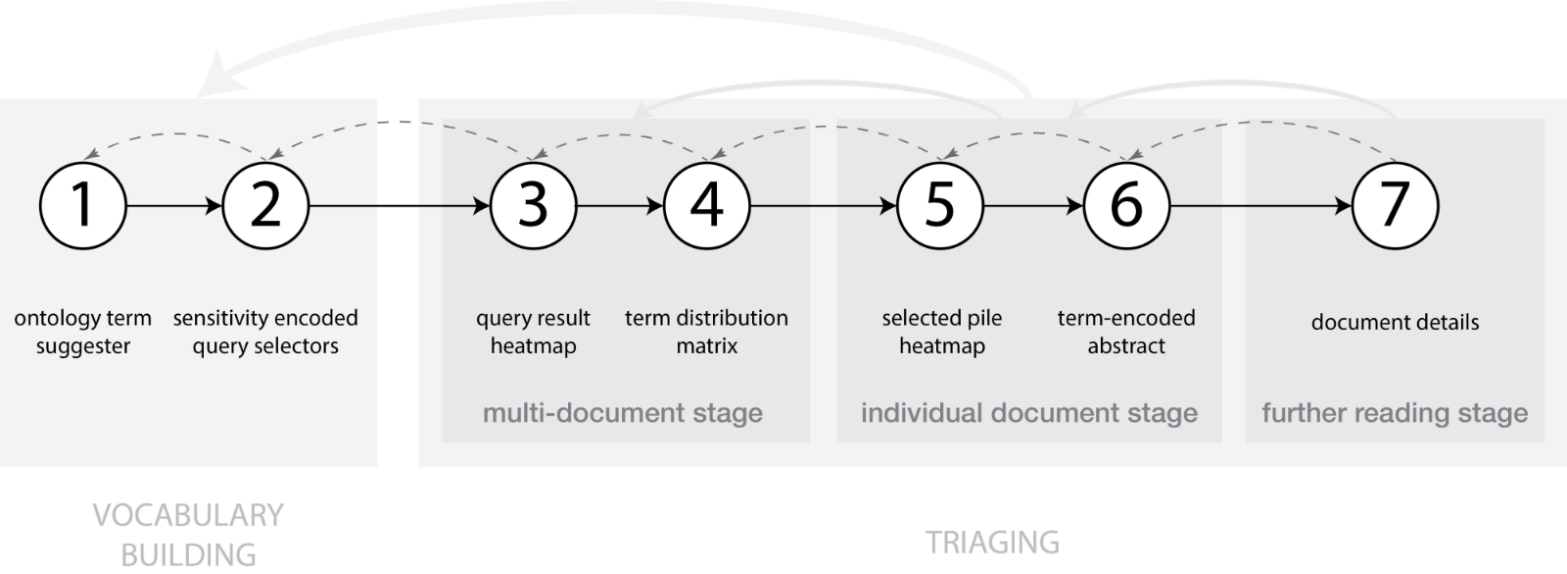
Overall search and triage process supported by OVERT-MED. Users take 7 main steps—the first 2 within a vocabulary building phase, and the next 5 within a triaging phase. OVERT-MED: Ontology-Driven Visual Search and Triage Interface for MEDLINE.

There are 7 main steps that users take when performing search and triaging tasks with OVERT-MED—the first 2 within a vocabulary building phase and the next 5 within a triaging phase. The triaging phase can be broken down into the 3 key stages. Figure 14 provides an overview of this process and shows the techniques we use to help users at each step. To help users build vocabulary and generate queries, we use an *ontology term suggester* and *sensitivity encoded query selectors*. After selecting a query, users move to the triaging phase, where they traverse through 3 stages. During the first stage—the multi-document stage—users are presented with a *query result heatmap* that encodes the appearance and frequency of query terms within the document result set. A keyboard interaction technique enables rapid navigation through the documents. To facilitate assessment at this stage, a *term distribution matrix* provides more information about each document within the heatmap. Together these techniques allow for rapid scanning to assess relevance and select documents for the next stage. During the second triaging stage—the individual document stage—users are presented with a *Selected Pile Heatmap* that encodes only the selected documents from the previous stage. As users browse the heatmap, they can inspect a *term-encoded abstract* of each individual document. The term-encoding supports quick detection of the appearance of query terms within the document abstract. After assessing the relevance of individual documents, users select documents to move to the next stage. During the third triaging stage—the further reading stage—users focus on a single document by viewing details in depth. Here, the PubMed entry for a document can be retrieved directly within OVERT-MED or within a new browser tab. At any point in the overall activity, users can return to any step and continue from there, which supports the iterative and cyclical nature of search and triaging tasks.

### Validation

Ongoing formative evaluation suggests that the design features in OVERT-MED can mitigate the two problems mentioned above. We tested OVERT-MED with a small group of users who are not domain-experts, and our proposal to use a formal ontology to help users articulate their information needs does seem to be useful. As mentioned previously, different types of users are known to search the scientific literature, many of which are not domain experts. For example, pediatricians often try to identify abnormal phenotypes in patients before referring them to a clinical geneticist. However, because they are not domain experts, pediatricians may not have very extensive knowledge and vocabulary of phenotypes. Even if they search the literature to identify phenotype names (eg, via PubMed), they may still not find phenotypes that are related to one another. As another example, patients are known to search the literature to learn more about their own conditions. As they are not domain experts, patients could also benefit from having access to an ontology such as HPO to help them build domain-specific knowledge and vocabulary. Thus, testing with users who are not domain experts can give an indication of the usefulness of our design strategies.

In our testing, we noticed that although an ontology can help users develop more appropriate vocabulary, users do not necessarily develop a good understanding of the ontology itself. As a robust mental model of the ontology may lead to even better search performance (eg, by knowing which entities are highly connected to others, knowing relationships among entities at multiple levels of abstraction, and so on), we have decided to pursue a solution to this as future work (see Future Work section). In addition, our multistage triaging shows promise in mitigating the information overload problem. Users were able to go back and forth through the triaging stages to satisfy information needs without being overwhelmed by long lists of documents.

### Limitations

There is 1 current limitation of OVERT-MED that should be noted: the MEDLINE data are limited to metadata and abstract text only, and do not include full texts. This is simply because the NLM does not release full-texts due to copyright issues. There is little we can do to address this issue. Empirical evidence, however, does suggest that the document title and abstract are among the most important features of a document in determining its relevance [37], so perhaps it is not a critical limitation.

### Future Work

We envision at least three lines of valuable future research:

First, developing interactive visualization techniques to support ontology sensemaking. The intention behind the current version of OVERT-MED is to help address the common problem of lack of adequate vocabulary. Although OVERT-MED appears to support users in improving their search terms and potentially developing some domain knowledge, it does not necessarily support users in making sense of the ontology itself—that is, understanding its size, organization, types of relationships, significant and insignificant entities, and so on. Interactive visualizations of ontologies may enhance search and triaging activities. Second, testing OVERT-MED with different ontologies in different contexts. This will help assess the transferability of the design features of OVERT-MED. Third, conducting formal testing of OVERT-MED. Although our informal testing has been useful, more formal testing will provide validation of the design strategies.

### Conclusions

We have developed a Web-based interactive visualization tool, OVERT-MED, to address two common problems in exploratory search—namely, the lack of adequate vocabulary to construct useful queries and the difficulty of dealing with very large result sets. The novelty of our approach is in the combination of (1) using an ontology to help build domain-specific knowledge and vocabulary when users need to be kept “in the loop” and (2) providing multistage triaging support using interactive visualizations to help mitigate the information overload problem. We anticipate these ideas can be applied successfully in other contexts where either of these issues exists.

## Abbreviations

HPO: Human Phenotype Ontology
MEDLINE: Medical Literature Analysis and Retrieval System Online
MeSH: Medical Subject Header
NLM: National Library of Medicine
OVERT-MED: Ontology-Driven Visual Search and Triage Interface for MEDLINE

## References

[1] Krupski TL, Dahm P, Fesperman SF, Schardt CM (2008). How to perform a literature search. J Urol.

[2] Islamaj DR, Murray GC, Névéol A, Lu Z (2009). Understanding PubMed user search behavior through log analysis. Database (Oxford).

[3] Kritz M, Gschwandtner M, Stefanov V, Hanbury A, Samwald M (2013). Utilization and perceived problems of online medical resources and search tools among different groups of European physicians. J Med Internet Res.

[4] Hersh WR, Crabtree MK, Hickam DH, Sacherek L, Friedman CP, Tidmarsh P, Mosbaek C, Kraemer D (2002). Factors associated with success in searching MEDLINE and applying evidence to answer clinical questions. J Am Med Inform Assoc.

[5] Parsons P, Sedig K, Mercer R, Khordad M, Knoll J, Rogan P (2015). Visual analytics for supporting evidence-based interpretation of molecular cytogenomic findings. Proceedings of the 2015 Workshop on Visual Analytics in Healthcare.

[6] Palotti J, Hanbury A, Müller H, Kahn C (2015). How users search and what they search for in the medical domain. Inf Retrieval J.

[7] Marchionini G (2006). Exploratory search: from finding to understanding. Communications of the ACM - Supporting exploratory search.

[8] Hersh WR, Hickam DH (1998). How well do physicians use electronic information retrieval systems? A framework for investigation and systematic review. J Am Med Assoc.

[9] Cui L, Carter R, Zhang G (2014). Evaluation of a novel conjunctive exploratory navigation interface for consumer health information: a crowdsourced comparative study. J Med Internet Res.

[10] Pang PC, Chang S, Verspoor K, Pearce J (2016). Designing health websites based on users' web-based information-seeking behaviors: a mixed-method observational study. J Med Internet Res.

[11] Ely JW, Osheroff JA, Chambliss ML, Ebell MH, Rosenbaum ME (2005). Answering physicians' clinical questions: obstacles and potential solutions. J Am Med Inform Assoc.

[12] Davies K, Harrison J (2007). The information-seeking behaviour of doctors: a review of the evidence. Health Info Libr J.

[13] Dietze H, Alexopoulou D, Alvers MR, Barrio-Alvers L, Andreopoulos B, Doms A, Hakenberg J, Mönnich J, Plake C, Reischuck A, Royer L, Wächter T, Zschunke M, Schroeder M (2009). GoPubMed: Exploring PubMed with Ontological Background Knowledge. Bioinformatics for Systems Biology.

[14] NCBI.NLM.

[15] Furnas GW, Landauer TK, Gomez LM, Dumais ST (1987). The vocabulary problem in human-system communication. Commun ACM.

[16] Belkin NJ (2000). Helping people find what they don't know. Commun ACM.

[17] Patrick TB, Monga HK, Sievert ME, Houston HJ, Longo DR (2001). Evaluation of controlled vocabulary resources for development of a consumer entry vocabulary for diabetes. J Med Internet Res.

[18] Plovnick RM, Zeng QT (2004). Reformulation of consumer health queries with professional terminology: a pilot study. J Med Internet Res.

[19] Sievert M, Patrick T, Reid J (2001). Need a bloody nose be a nosebleed? or, lexical variants cause surprising results. Bull Med Libr Assoc.

[20] Zeng QT, Tse T (2006). Exploring and developing consumer health vocabularies. J Am Med Inform Assoc.

[21] Lowe HJ, Barnett GO (1994). Understanding and using the medical subject headings (MeSH) vocabulary to perform literature searches. J Am Med Assoc.

[22] Malhotra A, Gündel M, Rajput AM, Mevissen H, Saiz A, Pastor X, Lozano-Rubi R, Martinez-Lapiscina EH, Martinez-Lapsicina EH, Zubizarreta I, Mueller B, Kotelnikova E, Toldo L, Hofmann-Apitius M, Villoslada P (2015). Knowledge retrieval from PubMed abstracts and electronic medical records with the Multiple Sclerosis Ontology. PLoS One.

[23] Lu Z (2011). PubMed and beyond: a survey of web tools for searching biomedical literature. Database (Oxford).

[24] Hoeber O, Khazaei T (2015). Evaluating citation visualization and exploration methods for supporting academic search tasks. Online Information Review.

[25] Hoeber O (2014). Visual Search Analytics: Combining Machine Learning and Interactive Visualization to Support Human-Centred Search.

[26] Hearst M, Elliott A, English J, Sinha R, Swearingen K, Yee K (2002). Finding the flow in web site search. Commun ACM.

[27] Yee K, Swearingen K, Li K, Hearst M (2003). Faceted metadata for image search and browsing.

[28] Dork M, Williamson C, Carpendale S (2009). Towards Visual Web Search?: Interactive Query Formulation and Search Result Visualization.

[29] Diriye A, Tombros A, Blandford A (2012). A Little Interaction Can Go a Long Way: Enriching the Query Formulation Process. Lect Notes Comput Sci.

[30] Joho H, Coverson C, Sanderson M, Beaulieu M (2002). Hierarchical presentation of expansion terms.

[31] Gruber T (1991). The role of common ontology in achieving sharable, reusable knowledge bases.

[32] Chandrasekaran B, Josephson JR, Benjamins VR (1975). What are ontologies, and why do we need them?. IEEE Intell Syst.

[33] Guarino N, Oberle D, Staab S (2009). What Is an Ontology?. Handbook on Ontologies.

[34] Robinson PN, Köhler S, Bauer S, Seelow D, Horn D, Mundlos S (2008). The Human Phenotype Ontology: a tool for annotating and analyzing human hereditary disease. Am J Hum Genet.

[35] Mavri A, Loizides F, Photiades T, Zaphiris P (2013). We Have the Content…Now What?: The role of Structure and Interactivity in Academic Document Triage Interfaces. Inf Des J.

[36] Loizides F, Buchanan G (2013). Towards a Framework for Human (Manual) Information Retrieval. Multidisciplinary Information Retrieval.

[37] Loizides F, Buchanan G (2009). An empirical study of user navigation during document triage.

[38] Khazaei T, Hoeber O (2016). Supporting academic search tasks through citation visualization and exploration. Int J Digit Libr.

[39] Spink A, Wolfram D, Jansen M, Saracevic T (2001). Searching the web: the public and their queries. J Am Soc Inf Sci.

[40] Silverstein C, Marais H, Henzinger M, Moricz M (1999). Analysis of a very large web search engine query log. SIGIR Forum.

[41] Scaife M, Rogers Y (1996). External cognition: how do graphical representations work?. Int J Hum Comput Stud.

[42] Larkin J, Simon H (1987). Why a diagram is (sometimes) worth ten thousand words. Cogn Sci.

[43] Hearst M (1995). TileBars: Visualization of Term Distribution Information in Full Text Information Access.

[44] Gorg C, Liu Z, Stasko J (2013). Reflections on the evolution of the Jigsaw visual analytics system. Inf Vis.

[45] Thomas W, Alexopoulou D, Dietze H, Schroeder M (2009). Searching biomedical literature with anatomy ontologies. Anatomy Ontologies for Bioinformatics.

[46] Wei C, Kao H, Lu Z (2013). PubTator: a web-based text mining tool for assisting biocuration. Nucleic Acids Res.

[47] Muin M, Fontelo P, Liu F, Ackerman M (2005). SLIM: an alternative Web interface for MEDLINE/PubMed searches - a preliminary study. BMC Med Inform Decis Mak.

[48] Eaton AD (2006). HubMed: a web-based biomedical literature search interface. Nucleic Acids Res.

[49] Wang J, Cetindil I, Ji S, Li C, Xie X, Li G, Feng J (2010). Interactive and fuzzy search: a dynamic way to explore MEDLINE. Bioinformatics.

[50] Yu H, Kim T, Oh J, Ko I, Kim S (2009). RefMed: relevance feedback retrieval system fo PubMed.

[51] Lu Z (2011). PubMed and beyond: a survey of web tools for searching biomedical literature. Database (Oxford).

[52] Salton G, Buckley C (1988). Term-weighting approaches in automatic text retrieval. Inf Process Manag.

[53] Salton G, Wong A, Yang CS (1975). A vector space model for automatic indexing. Commun ACM.

[54] Bostock M, Ogievetsky V, Heer J (2011). D³: data-driven documents. IEEE Trans Vis Comput Graph.

[55] Spence R, Tweedie L (1998). The Attribute Explorer: information synthesis via exploration. Interact Comput.

[56] Spence R (2002). Sensitivity encoding to support information space navigation: a design guideline. Inf Vis.

[57] Wilkinson L, Friendly M (2009). The History of the Cluster Heat Map. Am Stat.

[58] Hoeber O, Yang X (2009). HotMap: supporting visual exploration of web search results. J Am Soc Inf Sci.

